# Treatment Patterns and Outcomes of Elderly Patients With Potentially Curable Esophageal Cancer

**DOI:** 10.3389/fonc.2022.778898

**Published:** 2022-02-14

**Authors:** Yang Yang, Mengyuan Chen, Jiping Xie, Yongling Ji, Liming Sheng, Guoqin Qiu, Xianghui Du, Qichun Wei

**Affiliations:** ^1^ Department of Radiation Oncology, The Second Affiliated Hospital, Zhejiang University School of Medicine, Hangzhou, China; ^2^ Department of Thoracic Radiotherapy, The Cancer Hospital of the University of Chinese Academy of Sciences (Zhejiang Cancer Hospital), Institute of Cancer and Basic Medicine (IBMC), Chinese Academy of Sciences, Hangzhou, China; ^3^ Zhejiang Key Laboratory of Radiation Oncology, The Cancer Hospital of the University of Chinese Academy of Sciences (Zhejiang Cancer Hospital), Institute of Basic Medicine and Cancer (IBMC), Chinese Academy of Sciences, Hangzhou, China; ^4^ Department of Radiation Oncology, Yuyao People’s Hospital, Ningbo, China

**Keywords:** esophageal cancer, treatment patterns, surgery, chemoradiotherapy, SEER

## Abstract

**Objectives:**

The proportion of elderly patients with esophageal cancer (EC) is increasing due to prolonged life expectancy and aging process. The aim of the study is to explore the optimal treatment strategy for elderly patients (aged ≥70 years) with locally advanced EC.

**Methods:**

Eligible patients with cT2-4aNxM0 EC were identified in the Surveillance, Epidemiology, and End Results database from 2010 to 2016. Treatment patterns were divided into six groups: surgical resection (S), chemoradiotherapy (CRT), trimodality therapy (CRT+S), radiotherapy (RT), chemotherapy (CT), or observation with no treatment (Obs). Survival between groups was compared using the log-rank test, and the Cox proportional hazards model was used to identify factors associated with overall survival (OS).

**Results:**

A total of 2917 patients with potentially curable EC were identified. Of all the patients included, 6.7%, 51.8%, 18.0%, 9.4% and 3.6%received S, CRT, CRT+S, RT, and CT, respectively, whereas 10.6% underwent Obs. The 3-year OS estimates were 30.2% (95% confidence interval [CI]: 23.5–38.9%), 25.4% (95% CI: 22.8–28.3%),44.3% (95% CI: 39.3–49.9%), 11.4% (95% CI: 7.7–17.0%), 16.1% (95% CI: 9.1–28.3%), and 5.6% (95% CI: 3.2–9.8%) for S, CRT, CRT+S RT, CT, and Obs (p<0.001), respectively. Overall, patents underwent CRT+S had the longest OS, compared to other treatment patterns, and the survival difference was not significant between patients receiving CRT and S (p=0.12) in the elderly population. However, the survival benefits of trimodality therapy over CRT gradually weakened with the increase in age, and became statistically non-significant for EC patients aged ≥80 years (p=0.35). Multivariate analysis showed that treatment patterns, age, sex, tumor grade, T stage, N stage, and marital status were significantly associated with OS.

**Conclusion:**

Generally, the use of trimodality therapy was associated with the longest OS, the survival benefits were comparable between CRT and S alone, and CRT was superior to RT or CT alone in elderly patients with curable EC. For patients intolerable to surgery or aged ≥80 years, definitive CRT should be considered as a preferable option.

## Introduction

Esophageal cancer (EC) is one of the most common cancers worldwide. Moreover, 604,100 people were newly diagnosed with EC, whereas 544,076 people died of EC in 2020, according to Global Cancer Statistics (1). The peak age of EC incidence is between 60 and 70 years, and then, the incidence of EC decreases with age. According to the Global Burden of Disease report, approximately 30% of all newly diagnosed patients with EC are older than 70 years (2). The proportion of elderly patients with EC intends to increase gradually in the future due to prolonged life expectancy and aging process. However, evidence concerning treatment strategies in elderly patients with EC is still inadequate, since most data are from clinical trials with younger patients, in which the elderly have been often neglected and underrepresented (3).

Since the publication of the CROSS study (4), neoadjuvant chemoradiotherapy (CRT) followed by esophagectomy is recommended for patients with potentially curable EC, according to the National Comprehensive Cancer Network and other guidelines (5). However, elderly patients tend to have poorer performance scores, more multiple comorbidities, and shorter life expectancy compared to young individuals. Moreover, they might be less tolerant to esophagectomy or definitive CRT, due to severe complications and side effects (6–8). Thus, treatment for elderly patients with esophageal carcinoma appears to be underutilized (9). Controversies on the selection of the optimal treatment strategy for elderly patients with curable EC, including esophagectomy versus CRT (10, 11) or CRT versus radiotherapy (RT) alone, still continue (8, 12). For example, Abrams JA et al. have reported that esophagectomy may be associated with better survival for early-stage EC patients aged ≥65 years compared to CRT (11), whereas Koeter M et al. have found that survival was comparable among elderly patients (aged ≥75 years) with esophageal squamous cell carcinoma (ESCC) who underwent surgery or received definitive CRT (10). However, most previous studies have included a relatively small number of samples or only compared the efficiency of two main treatment patterns (10, 11), with inconsistent definitions of “elderly population” from aged 65 years and older (11, 13) to ≥70 (12, 14) or 75 years (10) or more than 80 years (15). Given the conflicting and insufficient data on this population, the optimal treatment strategy for elderly patients with potentially curable EC needs to be further investigated.

In the present study, we systematically evaluated all treatment patterns and outcomes of elderly patients with potentially curable EC using the Surveillance, Epidemiology, and End Results (SEER) database. To provide a comprehensive understanding of the impact of age on treatment selections and survival outcomes, patients were further divided into four age groups as follows, age 70–74, 75–79, 80–84, and ≥85 years.

## Materials and Methods

### Patient Selection

#### Data Source

This study involved extraction of eligible patient-level data on elderly EC cases from the SEER database, which collects data on cancer incidence, treatment, and survival from population-based cancer registries, covering 26% of the US population (16). In our study, elderly patients referred to those aged 70 years or older, mainly according to the definitions of elderly patients with NSCLC (17, 18). To reflect the modern radiation technology (intensity-modulated RT) and recent progress in EC treatment, patients aged ≥70 years diagnosed with stage T2-T4aNxM0 EC from 2010 to 2016 were identified from the SEER database, using SEER*Stat software (version 8.3.8, NIH, USA).

#### Inclusion and Exclusion Criteria

Potentially curable esophageal cancer in our study was recognized as localized disease without distant metastases, which can be treated by radical surgery or definitive CRT. The inclusion criteria were as follows: (1) patients with histologically confirmed EC, esophageal adenocarcinoma (EAC), or ESCC; (2) patients aged >70 years; (3) patients with stage T2-T4aNxM0, according to the guidelines of the American Joint Committee on Cancer 7th edition; (4) patients with treatment information, including surgery, radiation sequence with surgery, and chemotherapy (CT).The exclusion criteria were as follows: (1) patients who underwent endoscopic resection; (2) patients receiving resection through biopsy or regional lymph node aspiration; and (3) patients with missing or incomplete data on treatment information, including RT or survival status.

### Study Definitions

In our study, treatment patterns for elderly patients were divided into six groups: surgical resection (S), CRT, CRT+S, RT, CT, or observation with no treatment (Obs). The treatment definitions were as follows: the treatment of surgery was defined as patients who underwent esophagectomy alone or combined with RT or CT, whereas CRT referred to patients receiving RT with CT, concurrent or sequential. CRT+S referred to patients receiving CRT before or after surgery, RT or CT was defined as patients receiving RT or CT alone. The primary endpoint in our study was overall survival (OS), and the secondary endpoint was cancer-specific survival (CSS), which was defined as the intervals between the date of diagnosis and the occurrence of any-cause or cancer-specific death, respectively.

### Statistical Analyses

Differences in baseline characteristics between patients treated with different patterns were compared using Pearson’s chi-squared test or Fisher’s exact test. Multinomial logistic regression was used to determine the predictors of the use of trimodality therapy (CRT+S). Survival was estimated using the Kaplan-Meier method, and the log-rank test was used to compare survival curves. Univariate Cox regression analysis was performed to identify the significant variables associated with survival, and variables with a p value less than 0.10 were included in the multivariate Cox model. P for the interaction between subgroup analyses was calculated using the likelihood ratio test. All tests were two-sided, and p values <0.05 were considered statistically significant. Statistical analysis including Pearson’s chi-squared test, logistic regression was performed by the International Business Machines (IBM) Statistical Package for the Social Sciences statistics software (version 22.0; IBM Corp., Armonk, NY, USA). R version 3.4.1, including ggplot2, survival, survminer, foreign, rms packages, was used for Cox regression analysis, Kaplan-Meier survival curve comparison and nomgram drawing.

## Results

### Patient Characteristics and Patterns of Care

**Figure 1:**
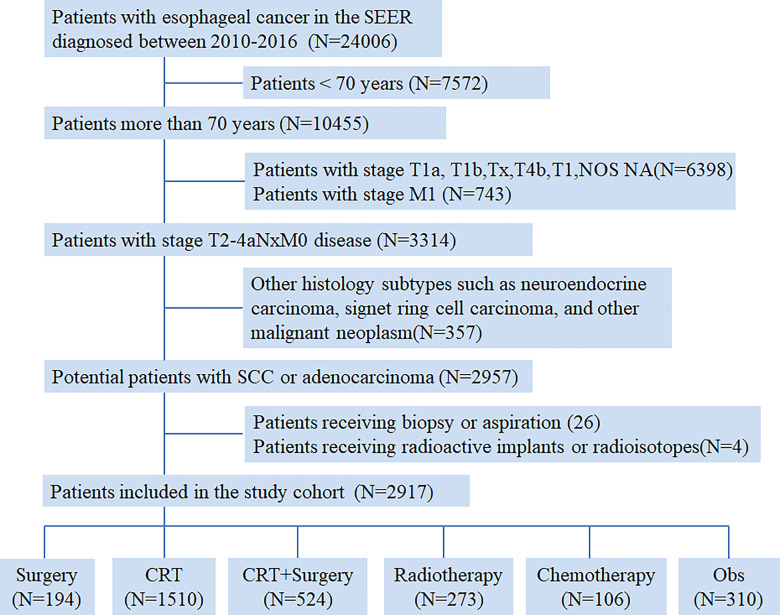
Flow chart of patient selection from the Surveillance, Epidemiology, and End Results (SEER) database.

**Figure 2:**
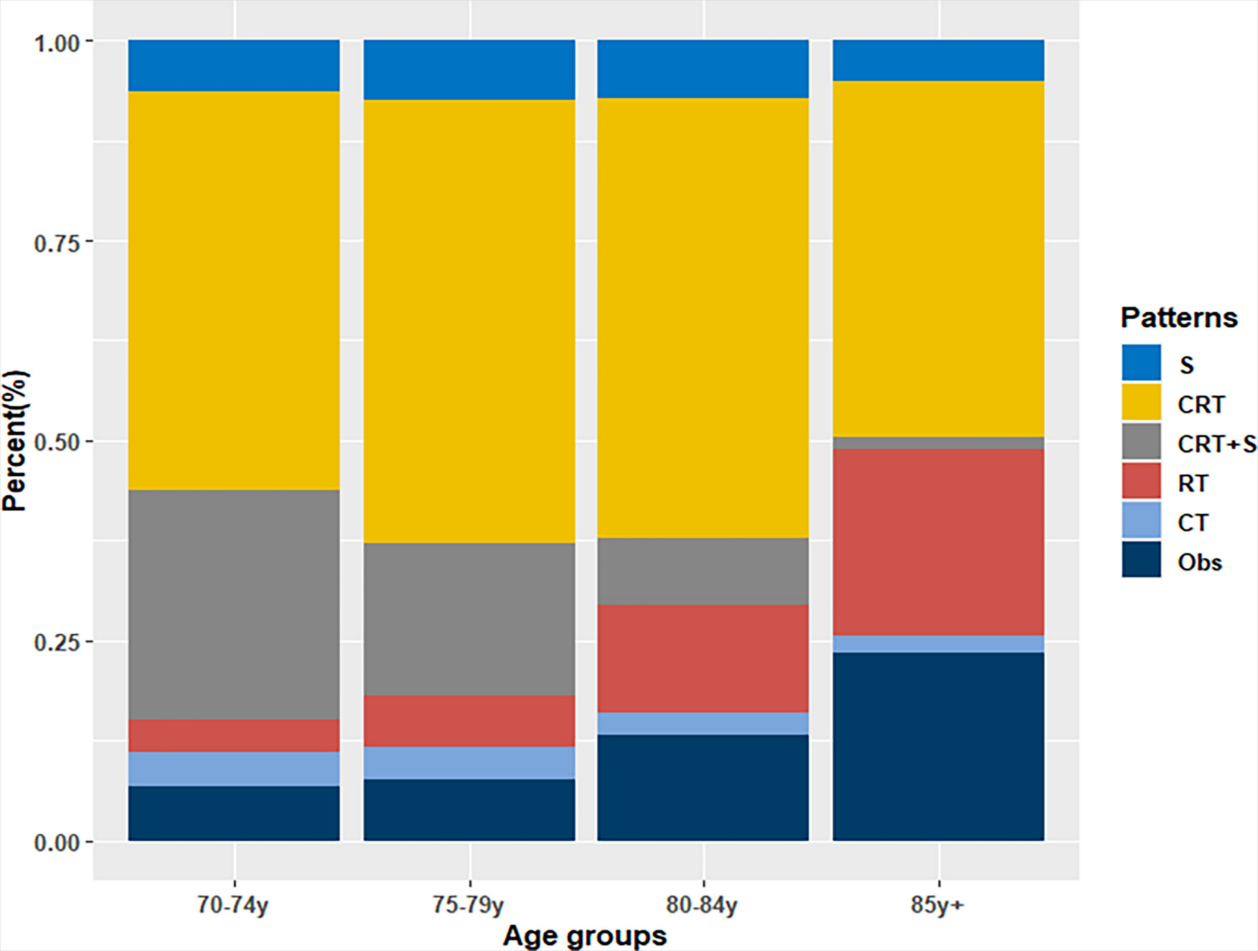
Treatment patterns of elderly patients with EC by age group.

From 2010 to 2016, a total of 24006 newly diagnosed patients with EC were found in the SEER database; 43.55% (N=10455) of them were elderly patients aged > 70 years. According to the inclusion criteria, 2917 patients with potentially curable EC were identified in our study. Of all the patients included, 6.7% (n =194) received surgery alone, more than one half patients (N=1510, 51.8%) received CRT, 18.0% (N=524) received CRT+S, 9.4% (N=273) received RT, and 3.6% (N=106) received CT, whereas 10.6% (N=310) underwent Obs. A flowchart of patient selection was presented in **Figure 1**. Data were widely collected for each patient for analysis, including patient characteristics, clinicopathologic tumor parameters, treatment, and survival information. Baseline patient demographics and clinical characteristics are listed in **Table 1**. As shown in **Figure 2**, patients receiving CRT+S tended to be younger, and more patients underwent RT alone or Obs, with the increase in age (p<0.001). Other variables significantly associated with trimodality therapy included earlier T stage (odds ratio [OR] for T2 = 6.01, 95% confidence interval[CI]:2.62-1.78, p<0.001; OR for T3 = 6.12, 95%CI:3.29-11.60, p<0.001), married status(OR=2.58, 95%CI:1.26-5.30, p=0.01), middle or lower third location of primary lesions (OR for middle location=2.93, 95%CI:1.47-5.87, p<,0.001; OR for lower location=3.53, 95% CI: 1.98–6.28, p<0.001) and white race(OR=2.8, 95%CI:1.23-6.36, p=0.014).

**Table 1:** Baseline Patient Demographics and Clinical and Tumor Characteristics.

Variables	Total N = 2917 (100%)	S N = 194 (6.7%)	CRT N = 1510 (51.8%)	CRT+S N = 524 (18.0%)	RT N = 273 (9.4%)	CT N = 106 (3.6%)	Obs N = 310 (10.6%)	P value
**Age at diagnosis**								<0.001
70-74 years	1081 (37.1%)	68 (35.1%)	540 (35.8%)	309 (59.0%)	45 (16.5%)	46 (43.4%)	73 (23.5%)	
75-79 years	836 (28.7%)	62 (32.0%)	463 (30.7%)	160 (30.5%)	53 (19.4%)	35 (33.0%)	63 (20.3%)	
80-84 years	591 (20.3%)	43 (22.2%)	325 (21.5%)	49 (9.4%)	80 (29.3%)	16 (15.1%)	78 (25.2%)	
85+ years	409 (14.0%)	21 (10.8%)	182 (12.1%)	6 (1.1%)	95 (34.8%)	9 (8.5%)	96 (31.0%)	
**Sex**								<0.001
Male	2170 (74.4%)	136 (70.1%)	1118 (74.0%)	442 (84.4%)	194 (71.1%)	78 (73.6%)	202 (65.2%)	
Female	747 (25.6%)	58 (29.9%)	392 (26.0%)	82 (15.6%)	79 (28.9%)	28 (26.4%)	108 (34.8%)	
**Race**								<0.001
White	2601 (89.2%)	178 (91.8%)	1339 (88.7%)	497 (94.8%)	232 (85.0%)	90 (84.9%)	265 (85.5%)	
Black	173 (5.9%)	5 (2.6%)	100 (6.6%)	16 (3.1%)	21 (7.7%)	7 (6.6%)	24 (7.7%)	
Others	143 (4.9%)	11 (5.7%)	71 (4.7%)	11 (2.1%)	20 (7.3%)	9 (8.5%)	21 (6.8%)	
**Histological subtype**								<0.001
Adenocarcinoma	1108 (38.0%)	67 (34.5%)	661 (43.8%)	97 (18.5%)	129 (47.3%)	39 (36.8%)	115 (37.1%)	
Squamous cell	1809 (62.05)	127 (65.5%)	849 (56.2%)	427 (81.5%)	144 (52.7%)	67 (63.2%)	195 (62.9%)	
**Stage**								<0.001
II	1404 (48.1%)	115 (59.3%)	736 (48.7%)	201 (38.4%)	164 (60.1%)	40 (37.7%)	148 (47.7%)	
III	1513 (51.9%)	79 (40.7%)	774 (51.3%)	323 (61.6%)	109 (39.9%)	66 (62.3%)	162 (52.3%)	
**T stage**								<0.001
T2	740 (25.4%)	65 (33.5%)	394 (26.1%)	116 (22.1%)	69 (25.3%)	22 (20.8%)	74 (23.9%)	
T3	1959 (67.2%)	122 (6.2%)	1030 (68.2%)	385 (73.5%)	185 (67.8%)	71 (67.0%)	166 (53.5%)	
T4a	218 (7.5%)	7 (3.6%)	86 (5.7%)	23 (4.4%)	19 (7.0%)	13 (12.2%)	70 (22.6%)	
**N stage**								<0.001
N0	1259 (43.2%)	104 (53.6%)	624 (41.3%)	164 (31.3%)	158 (57.9%)	39 (36.8%)	170 (54.8%)	
N1	1285 (44.1%)	47 (24.2%)	696 (46.1%)	278 (53.1%)	90 (7.0%)	54 (50.9%)	120 (38.7%)	
N2	310 (10.6%)	28 (14.4%)	166 (11.0%)	71 (13.5)	21 (7.7%)	10 (9.4%)	310 (10.6%)	
N3	63 (2.2%)	15 (7.7%)	24 (1.6%)	11 (2.1%)	4 (1.5%)	3 (2.8%)	63 (2.2%)	
**Grade**								<0.001
Well (G1)	170 (5.8%)	11 (5.7%)	86 (5.7%)	26 (5.0%)	17 (6.2%)	5 (4.7%)	25 (8.1%)	
Moderate (G2)	1094 (37.5%)	85 (43.8%)	573 (37.9%)	209 (39.9%)	93 (34.1%)	35 (33.0%)	99 (31.9%)	
Poorly (G3)	1124 (38.5%)	83 (42.8%)	548 (36.3%)	231 (44.1%)	110 (40.3%)	46 (43.4%)	106 (34.2%)	
Undifferentiated (G4)	26 (0.9%)	5 (19.2%)	13 (0.9%)	2 (0.4%)	1 (0.4%)	1 (0.9%)	4 (1.3%)	
Unknown	503 (17.2%)	10 (5.2%)	290 (19.2%)	56 (10.7%)	52 (19.0%)	19 (17.9%)	76 (24.5%)	
**Tumor location**								<0.001
Upper third	243 (8.3%)	15 (7.7%)	155 (10.3%)	9 (1.7%)	28 (10.3%)	8 (7.5%)	28 (9.0%)	
Middle third	602 (20.6%)	32 (16.5%)	360 (23.8%)	61 (11.6%)	79 (28.9%)	17 (16.0%)	53 (17.1%)	
Lower third	1841 (63.1%)	134 (69.1%)	879 (58.2%)	429 (81.9%)	149 (54.6%)	61 (57.5%)	189 (61.0%)	
Unknown	231 (7.9%)	13 (6.7%)	116 (7.7%)	25 (4.8%)	17 (6.2%)	20 (18.9%)	40 (12.9%)	
**Marital status**								<0.001
Married	1684 (57.7%)	96 (49.5%)	887 (58.7%)	372 (71.0%)	137 (50.2%)	65 (61.3%)	127 (41.0%)	
Unmarried	1087 (37.3%)	86 (44.3%)	554 (36.7%)	133 (25.4%)	122 (44.7%)	27 (25.5%)	165 (53.2%)	
Unknown	146 (5.0%)	12 (6.2%)	69 (4.6%)	19 (3.6%)	14 (5.1%)	14 (13.2%)\	18 (5.8%)	

### Survival Analyses

**Figure 3:**
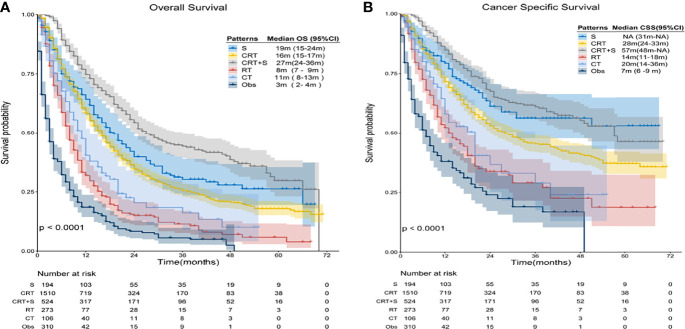
Kaplan-Meier survival curves of OS and CSS for elderly patients with potentially curable EC. **(A)** Overall Survival(OS), **(B)** Cancer Specific Survival (CSS).

In the overall analysis for OS, patients receiving trimodality therapy showed significantly better survival than patients who underwent other treatment patterns, whereas observation resulted in the worst survival, as shown in **Figure 3A**. The 3-year OS estimates were 30.2% (95% confidence interval [CI]: 23.5–38.9%), 25.4% (95% CI: 22.8–28.3%),44.3% (95% CI: 39.3–49.9%), 11.4% (95% CI: 7.7–17.0%), 16.1% (95% CI: 9.1–28.3%), and 5.6% (95% CI: 3.2–9.8%) for S, CRT, CRT+S RT, CT, and Obs (p<0.001), respectively. Compared to Obs, any treatment pattern was associated with superior OS, and the hazard ratios (HRs) for S, CRT, CRT+S, RT, and CT were 0.25(95%CI: 0.20-0.31), 0.29(95%CI:0.25-0.33), 0.17(95%CI:0.14-0.20), 0.54(95%CI:0.45-0.65) and 0.43(95%CI:0.33-0.56) (p<0.001), respectively. Further pairwise comparisons between groups showed that CRT+S significantly was related to better outcomes compared to any other treatment patterns (p<0.01), and no significant differences were observed between patients who underwent CRT and S alone(p=0.12). Moreover, patients receiving CRT had a significant survival advantage over RT or CT alone (p<0.01). For CSS, similar results were found (**Figure 3B**), and the 3-year CSS estimates were 56.3%(95%CI:47.7-66.4%), 45.3%(95%CI:41.8-49.1%),61.1%(95%CI:55.8-67.0%),29.0%(95%CI:21.5-39.0%), 28.9%(95%CI:17.5-47.7%) and 19.0%(95%CI:12.4-29.0%) for S, CRT, CRT+S, RT, CT, and Obs (p<0.001), respectively. In this analysis, the use of surgery or CRT+S brought better survival benefits than CRT (p=0.024,<0.001 respectively), indicating the critical role of surgery.

**Figure 4:**
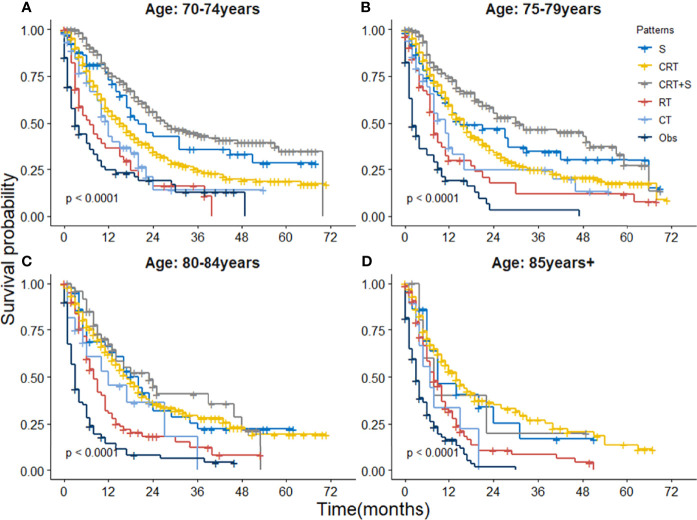
Kaplan-Meier survival curves of OS for elderly patients with potentially curable EC stratified by age group. **(A)** 70-74 year, **(B)** 75-79 years, **(C)** 80-84 years and **(D)** ≥85 years.

When further stratified by age group, the superiority of CRT+S was observed in almost all age groups, as shown in **Figure 4**. However, the survival benefits of CRT+S or surgery over CRT gradually weakened with the increase in age, and the 3 year-OS estimates were 20.6%(95%CI:11.8-36.0%) for S, 27.5%(95%CI:23.0-32.9%) for CRT and 38.5%(25.6-57.9%) for CRT+S respectively in EC patients aged >80 years, and the survival benefit of trimodality therapy was statistically non-significant (p=0.36 compared to S, 0.35 compared to CRT). The results of definitive CRT remained fairly stable over the age groups. Subgroup analyses stratified by other factors, including race, pathology, grade, stage, location, and marital status also supported the superiority of trimodality therapy and the results were presented in **Table S1**.

### Predictive Factors for OS in Elderly Patients With Esophageal Cancer

**Figure 5:**
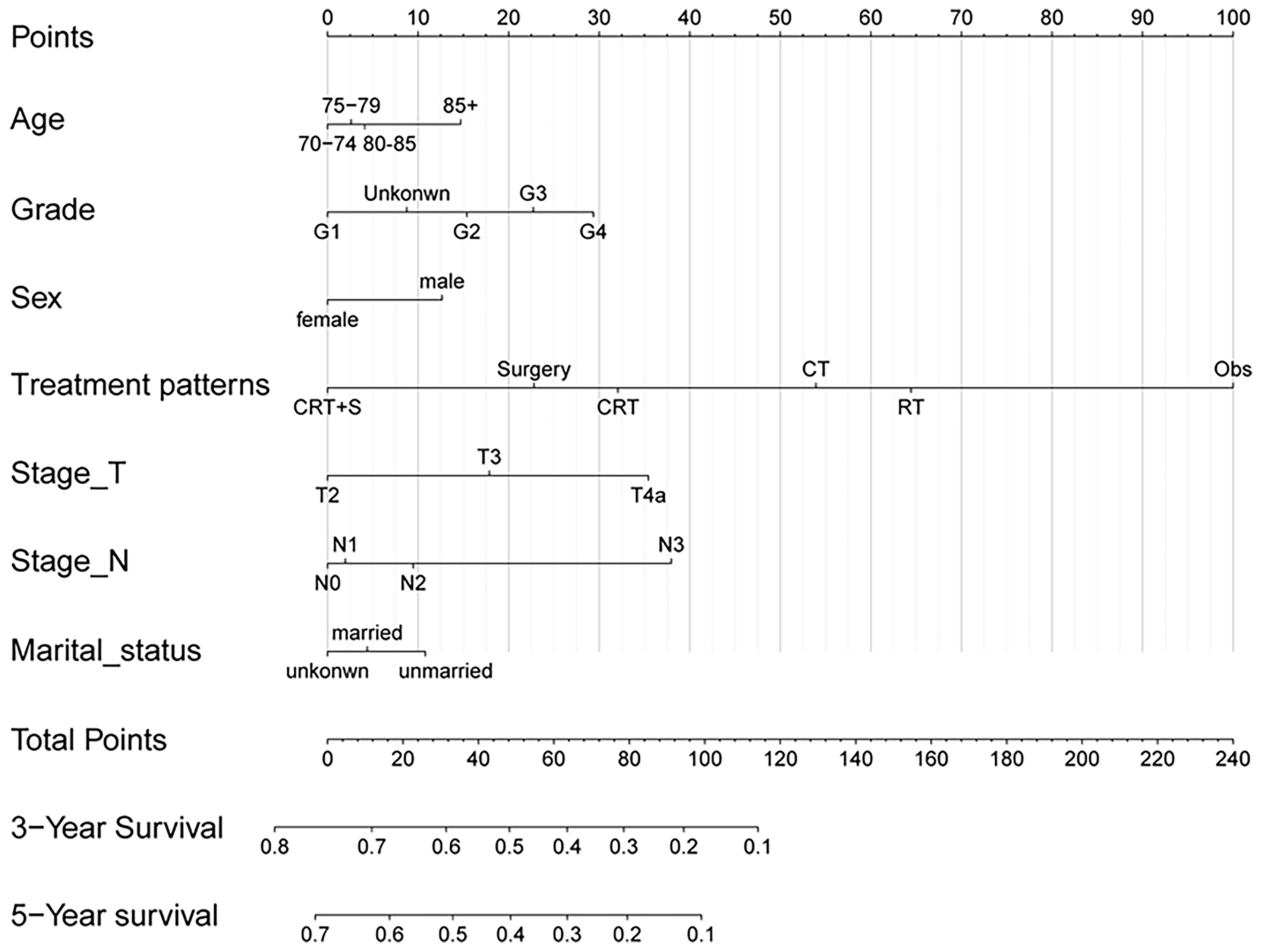
Nomogram for predicting 3- and 5-year probabilities of OS for elderly patients with potentially curable EC. The nomogram summed the points identified on the scale for each variable. The total points projected on the bottom scales indicate the probabilities of 3- and 5-year OS.

**Figure 6:**
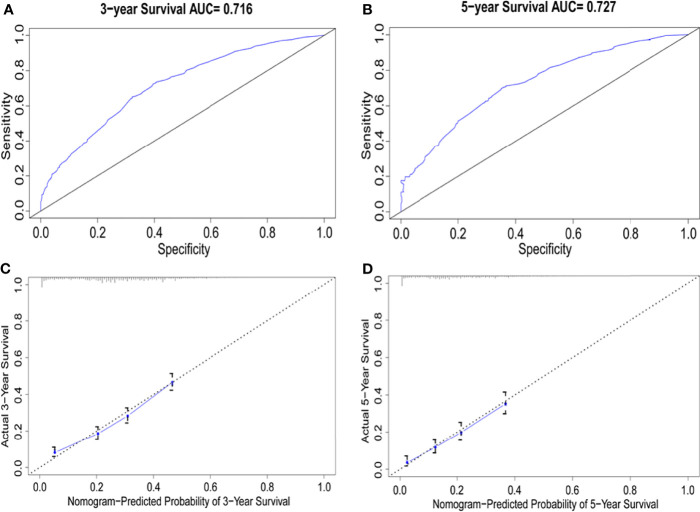
Comparison of the AUCs and Calibration curves for the nomogram. **(A, B)** Area under the curves of the two models to predict overall survival at 3 years **(A)** and 5 years **(B)**, **(C, D)** Calibration curves for the nomogram at 3 years **(C)** and 5 years**(D)**, the x axis represents the nomogram-predicted survival rate, whereas the y axis represents the actual survival rate.

Univariate Cox regression analysis was performed to identify the variables associated with OS in the selected cohort, and the significant predictive factors are consisted ofage, grade, sex, T stage, N stage, marital status, and treatment patterns, whilerace, pathological subtype, and tumor location were not significantly associated with OS in univariate analysis. The results were presented in **Table 2**. In multivariate Cox regression analysis, treatment pattern was still a statistically significant factor for improved OS (p<0.001). Other significant factors identified by multivariate analysis included age, sex, tumor grade, T stage, N stage, and marital status (p<0.05). Based on these predictive factors found on multivariate analysis, predictive nomograms were constructed to predict the 3- and 5-year cumulative incidence of OS for elderly patients with potentially curable EC (**Figure 5**), and the concordance index for the prediction of OS was calculated (0.68, 95% CI:0.66–0.70). In addition, the areas under the curve of the receiver operating characteristic curves for 3-year and 5-year OS were 0.72 and 0.73, respectively (**Figures 6A, B**). The calibration plots for the 3-year and 5-year cumulative probabilities of OS are presented in **Figures 6C, D**, which showed good consistency between nomogram prediction and actual observation.

**Table 2:** Univariate Analysis and Multivariate Cox regression analysis of OS in in elderly EC patients with ≥ 70 years.

Variables	Univariate Analysis		Multivariate analysis	
	HR with 95% CI	*p* value	HR with 95%CI	*p* value
**Age**				
70-74 years	1		1	
75-79 years	1.10 (0.98-1.23)	0.12	1.05 (0.93-1.18)	0.44
80-84 years	1.29 (1.13-1.46)	<0.001	1.07 (0.94-1.22)	0.28
85+ years	1.83 (1.60-2.11)	<0.001	1.29 (1.12-1.50)	0.001
**Sex**				
Male	1		1	
Female	0.90 (0.81-1.00)	0.05	0.80 (0.71-0.89)	<0.001
**Race**				
White	1			
Black	1.14 (0.94-1.37)	0.18		
Others	1.04 (0.84-1.29)	0.72		
**Pathology**				
Adenocarcinoma	1			
Squamous cell	1.03 (0.93-1.13)	0.58		
**Grade**				
Well (G1)	1		1	
Moderate (G2)	1.12 (0.90-1.39)	0.31	1.31 (1.06-1.63)	0.014
Poorly (G3)	1.31 (1.06-1.63)	0.012	1.50 (1.21-1.85)	<0.001
Undifferentiated (G4)	1.52 (0.90-2.54)	0.12	1.67 (0.99-2.81)	0.052
Unknown	1.19 (0.94-1.50)	0.14	1.17 (0.93-1.48)	0.18
**Stage**				
II	1		1	
III	1.25 (1.14-1.37)	<0.001	1.06 (0.86-1.31)	0.56
**T stage**				
T2	1		1	
T3	1.37 (1.23-1.54)	<0.001	1.34 (1.16-1.55)	<0.001
T4a	2.18 (1.81-2.61)	<0.001	1.77 (1.37-2.28)	<0.001
**N stage**				
N0	1		1	
N1	1.02 (0.92-1.13)	0.68	0.99 (0.84-1.18)	0.96
N2	1.10 (0.94-1.29)	0.24	1.13 (0.89-1.43)	0.32
N3	1.77 (1.31-2.38)	<0.001	1.85 (1.31-2.61)	0.001
**Tumor location**				
Upper third	1			
Middle third	0.94 (0.78-1.13)	0.52		
Lower third	0.98 (0.83-1.16)	0.81		
Unknown	1.11 (0.89-1.38)	0.37		
**Treatment patterns**				
Obs	1		1	
Surgery	0.25 (0.20-0.31)	<0.001	0.26 (0.20-0.32)	<0.001
CRT	0.29 (0.25-0.33)	<0.001	0.30 (0.26-0.35)	<0.001
CRT+S	0.17 (0.14-0.21)	<0.001	0.17 (0.14-0.20)	<0.001
RT	0.54 (0.45-0.65)	<0.001	0.54 (0.45-0.64)	<0.001
CT	0.43 (0.33-0.56)	<0.001	0.44 (0.34-0.58)	<0.001
**Marital status**				
Married	1		1	
Unmarried	1.21 (1.10-1.33)	<0.001	1.12 (1.01-1.24)	0.03
Unknown	1.06 (0.85-1.31)	0.62	0.93 (0.75-1.15)	0.49

## Discussion

In this large, population-based study, patterns of treatment and outcomes of elderly patients with potentially curable EC were comprehensively analyzed. The results showed that the usage ratios of trimodality therapy were decreased with the increase in age, CRT was mostly adapted in patients with locally advanced-stage EC, and RT alone was also occasionally employed, especially in patients aged ≥80 years. The survival analysis indicated that all the treatment patterns had survival benefits in elderly patients compared to Obs. The use of surgery or CRT_+S was associated with improved OS in the elderly EC patients, and CRT was superior to RT or CT alone. The results were stable across subgroup analyses stratified by most factors, including sex, clinical stage, histological subtype, and tumor location, demonstrating the reliability of our conclusions.

In younger patients, surgery-based trimodality therapy has been the standard treatment for locally advanced EC (19, 20). However, elderly patients tend to have a decline in physiological function and a high prevalence of chronic diseases, such as high blood pressure, diabetes, and cardiovascular system diseases, which make them difficult to respond to surgical trauma and recover slowly (21). Consequently, elderly patients undergoing esophagectomy for cancer are reported to have a significantly higher risk of postoperative mortality, especially in patients aged 75 years or older (7, 22, 23). Hence, elderly patients with EC should be cautiously evaluated and selected for surgery (24). In fact, only one-third of patients in our cohort underwent surgical resection, and the number of patients who underwent surgery decreased dramatically with increasing age. In this study, a significantly small number of patients (<10%) aged >80 years underwent surgery, reflecting concerns about postoperative morbidity and the underuse of surgery in elderly patients with EC.

In the survival analysis, elderly patients who underwent CRT+S lived significantly longer than those who received other treatment patterns, including CRT or surgery alone. The advantage of trimodality therapy was stable in both EAC and ESCC and across stages II to III.In consistence with our findings, a series of other retrospective studies also supported the use of surgery in elderly patients with EC, and esophagectomy was found to be associated with improved survival, even with increased risk of complications in elderly patients (6, 10, 11, 25, 26). In fact, it is also reported that trimodality therapy is a reasonable treatment option for properly selected elderly patients with EC, and can bring survival benefits (27). In addition to these findings, our study showed that the survival benefits of trimodality therapy or surgery disappeared in patients aged >80 years, while the benefit of definitive CRT remained fairly stable over the age groups, compared to RT or CT alone. Therefore, after comprehensive assessment and rigid screening, CRT+S should be preferentially recommended for elderly patients with good performance status and long expected life span, given the improved outcomes with treatment. For patients intolerable to surgery or aged >80 years, definitive CRT can be considered as an alternative option.

Considering postoperative morbidity and reduced quality of life, a large proportion of patients with EC favor non-operative treatment patterns. In our analysis, almost half of the patients chose CRT as their primary treatment, and 17.5% of patients aged >80 years only received RT alone. In the survival analysis, the survival benefit of CRT was only next to the trimodality therapy, and comparable with surgery alone, but remarkably superior to RT or CT alone. CRT has been the standard therapy for patients with locally advanced EC ineligible for surgery, since the Radiation Therapy Oncology Group 85-01 trial (28). However, in clinical practice, due to concerns regarding treatment-related adverse effects, including esophagitis, pneumonitis, and hematologic toxicity, part of elderly EC patients only undergo RT alone (29, 30). Several previous studies have demonstrated that definitive CRT might be considered as both effective and safe in elderly patients with EC, exhibiting similar long-term clinical benefits compared to younger patients (31–33). Our study once again confirmed the superiority of CRT over RT alone among all elderly age groups in a large population. RT alone should be recommended with caution even in the eldest group (aged >85 years). If patients cannot tolerate doublet CT combined with RT, single-drug oral chemotherapy drugs can be considered, such as S1, Xeloda, and other fluorouracil analogues (12, 34). Notably, a recent randomized phase 3 clinical trial led by our cancer center, confirmed that concurrent CRT with S-1 significantly improved 2-year OS compared with RT alone in older EC patients (35).

Our study has the following strengths. Firstly, our study used a population-based database with a large sample size and long-term follow-up period. Secondly, a comprehensive analysis of primary treatment patterns and wide-ranging subgroup analysis stratified by age, which made the conclusion reliable and stable, were performed in our study. However, our study also has several limitations. Firstly, as with any retrospective study, selection bias and unmeasured confounding variables are inevitable, and the baseline characteristics of patients in different patterns were not well balanced, which reflected real-world treatment choices. Secondly, some information was missing on patient characteristics and treatment process in the SEER database, such as performance status, radiation dose, CT regimens, and comorbidities, which limited the multivariate Cox regression analysis. Additionally, for study endpoints, data regarding recurrence and metastasis information were unavailable.

Given the natural limitations of retrospective studies, the findings of our study should be interpreted with caution in clinical practice. As described in the limitations of our study, selection bias should be considered. When selecting the optimal treatment pattern for elderly patients with EC, their physical conditions should be comprehensively assessed, including nutritional status, cardiopulmonary function, and associated underlying diseases. If possible, a comprehensive geriatric assessment (CGA) is recommended, which has been increasingly involved in guiding treatment decisions for elderly cancer patients (19). In younger patients with high CGA scores, more aggressive treatment options, such as surgery combined with neoadjuvant CRT, may be considered as the first option. For patients with higher age (aged ≥80 years) or poor general condition or unsuitable for surgery (regardless of medical reasons), CRT was the preferred treatment pattern.

## Conclusion

In this large sample population-based study, we found that curative-intent treatment patterns can provide survival benefits for elderly patients with EC. Trimodality therapy is associated with longest survival and thus should be considered as the first option, if it is feasible. Subsequently, CRT is remarkably superior to RT or CT alone in elderly patients with EC. For patients intolerable to surgery or aged ≥80 years, definitive CRT should be considered as a preferable selection. Age is not a restrictive condition for treatment options in elderly EC patients, and the optimal treatment strategy should take into account survival benefits and patient preferences in a multidisciplinary setting. Future clinical trials are needed to validate our findings and to reduce the occurrence of complications in the elderly population.

## Author’s Note

This manuscript was previously posted to Research Square: doi: 10.21203/rs.3.rs-443322/v1 (https://www.researchsquare.com/article/rs-443322/v1).

## Data Availability Statement

The original contributions presented in the study are included in the article/**Supplementary Material**. Further inquiries can be directed to the corresponding author.

## Author Contributions

QW and YY conceived and drafted the study. YY and MC collected and extracted the data. YY, MC, and JX designed the statistical analysis plan and performed the analyses. YY, YJ, and LS interpreted the results and prepared the manuscript. GQ and XD carefully revised the manuscript. All authors commented on drafts of the paper and approved the final manuscript.

## Funding

This work was supported by the fund of Zhejiang Province National Natural Science Foundation (No. LY20H160006) and Zhejiang Province Medical and Health Science and Technology Project (2020KY473 and 2022KY109).

## Conflict of Interest

The authors declare that the research was conducted in the absence of any commercial or financial relationships that could be construed as a potential conflict of interest.

## Publisher’s Note

All claims expressed in this article are solely those of the authors and do not necessarily represent those of their affiliated organizations, or those of the publisher, the editors and the reviewers. Any product that may be evaluated in this article, or claim that may be made by its manufacturer, is not guaranteed or endorsed by the publisher.

## Abbreviations

EC, esophageal cancer; SEER, the Surveillance, Epidemiology and End Results database; EAC, esophageal adenocarcinoma; ESCC, esophageal squamous cell carcinoma; S, surgical resection; CRT, chemoradiotherapy; RT, radiotherapy; CT, chemotherapy; Obs, observation with no treatment; OS, overall survival; CSS, cancer-specific survival; AUC, area under the curve; ROC, receiver operating characteristic curve; CGA, comprehensive geriatric assessment.
