# Supplementary material for: Treatment Patterns and Outcomes of Elderly Patients With Potentially Curable Esophageal Cancer
Source: Front Oncol. 2022 Feb 14;12:778898. doi: 10.3389/fonc.2022.778898 (PMC8882918; doi:10.3389/fonc.2022.778898)
Supplement: Supplementary file 1 [file DataSheet_1.docx]

**Table S1 Subgroup analysis of overall survival for elderly patients (≥70 years) with curable EC**

**in the SEER database from the year 2010 to 2016.**

| **Variables** | **HR with 95% CI,** *p* **value** | | | | | | | | | | | *p* **for interaction** |
| --- | --- | --- | --- | --- | --- | --- | --- | --- | --- | --- | --- | --- |
|  | **Obs** | **Surgery** | *p* | **CRT** | *p* | **CRT+S** | *p* | **RT** | *p* | **CT** | *p* |  |
| **Overall** | 1 | 0.25(0.20-0.31) | <0.001 | 0.29(0.25-0.33) | <0.001 | 0.17(0.14-0.20) | <0.001 | 0.54(0.45-0.65) | <0.001 | 0.43(0.33-0.56) | <0.001 |  |
| **Age**  70-74 years  75-79 years  80-84 years  85+ years | 1  1  1  1 | 0.30(0.20-0.46)  0.23(0.15-0.35)  0.26(0.17-0.40)  0.30(0.17-0.54) | <0.001  <0.001  <0.001  <0.001 | 0.42(0.32-0.55)  0.26(0.20-0.35)  0.26(0.20-0.34)  0.27(0.20-0.36) | <0.001  <0.001  <0.001  <0.001 | 0.23(0.17-0.31)  0.15(0.11-0.22)  0.22(0.14-0.34)  0.30(0.11-0.83) | <0.001  <0.001  <0.001  0.021 | 0.76(0.51-1.12)  0.44(0.29-0.67)  0.48(0.34-0.68)  0.50(0.37-0.68) | 0.20  <0.001  <0.001  <0.001 | 0.58(0.37-0.90)  0.37(0.23-0.60)  0.39(0.21-0.74)  0.58(0.29-1.17) | 0.016  <0.001  0.004  0.13 | 0.45 |
| **Sex**  Male  Female | 1  1 | 0.26(0.20-0.34)  0.24(0.16-0.26) | <0.001  <0.001 | 0.34(0.29-0.40)  0.20(0.15-0.26) | <0.001  <0.001 | 0.19(0.16-0.24)  0.10(0.07-0.16) | <0.001  <0.001 | 0.58(0.47-0.73)  0.47(0.34-0.65) | <0.001  <0.001 | 0.43(0.31-0.58)  0.45(0.28-0.73) | <0.001  0.001 | <0.001 |
| **Race**  White  Black  Others | 1  1  1 | 0.26(0.20-0.32)  0.11(0.03-0.38)  0.35(0.13-0.98) | <0.001  <0.001  0.047 | 0.29(0.25-0.34)  0.15(0.09-0.24)  0.51(0.28-0.94) | <0.001  <0.001  0.030 | 0.17(0.14-0.20)  0.12(0.05-0.26)  0.36(0.13-0.99) | <0.001  <0.001  0.047 | 0.56(0.46-0.68)  0.33(0.17-0.63)  0.62(0.29-1.34) | <0.001  0.001  0.23 | 0.40(0.30-0.54)  0.43(0.16-1.14)  0.95(0.40-2.28) | <0.001  0.09  0.91 | 0.38 |
| **Pathology**  Adenocarcinoma  Squamous cell | 1  1 | 0.19(0.13-0.28)  0.28(0.22-0.38) | <0.001  <0.001 | 0.20(0.16-0.25)  0.36(0.30-0.43) | <0.001  <0.001 | 0.12(0.08-0.17)  0.20(0.16-0.25) | <0.001  <0.001 | 0.38(0.29-0.50)  0.68(0.54-0.87) | <0.001  0.002 | 0.36(0.23-0.56)  0.47(0.34-0.64) | <0.001  <0.001 | 0.001 |
| **Grade**  G1-2  G3-4  Unknown | 1  1  1 | 0.25(0.18-0.35)  0.24(0.17-0.33)  0.10(0.03-0.33) | <0.001  <0.001  <0.001 | 0.27(0.22-0.33)  0.28(0.22-0.36)  0.32(0.24-0.44) | <0.001  <0.001  <0.001 | 0.15(0.12-0.20)  0.15(0.12-0.20)  0.24(0.15-0.39) | <0.001  <0.001  <0.001 | 0.52(0.40-0.70)  0.52(0.38-0.69)  0.58(0.39-0.86) | <0.001  <0.001  0.006 | 0.38(0.24-0.58)  0.48(0.33-0.71)  0.36(0.19-0.66) | <0.001  <0.001  0.001 | 0.65 |
| **Stage**  II  III | 1  1 | 0.19(0.14-0.27)  0.38(0.28-0.51) | <0.001  <0.001 | 0.28(0.23-0.34)  0.30(0.25-0.36) | <0.001  <0.001 | 0.17(0.13-0.22)  0.16(0.13-0.20) | <0.001  <0.001 | 0.61(0.48-0.78)  0.48(0.37-0.63) | <0.001  <0.001 | 0.36(0.23-0.55)  0.46(0.33-0.63) | <0.001  <0.001 | 0.005 |
| **T stage**  T2  T3  T4a | 1  1  1 | 0.16(0.10-0.26)  0.36(0.27-0.47)  0.24(0.10-0.60) | <0.001  <0.001  0.002 | 0.27(0.20-0.36)  0.35(0.29-0.42)  0.20(0.14-0.29) | <0.001  <0.001  <0.001 | 0.20(0.14-0.29)  0.19(0.15-0.24)  0.10(0.05-0.20) | <0.001  <0.001  <0.001 | 0.62(0.42-0.91)  0.60(0.47-0.75)  0.47(0.27-0.80) | 0.014  <0.001  0.006 | 0.39(0.21-0.71)  0.46(0.33-0.64)  0.51(0.28-0.96) | 0.002  <0.001  0.035 | 0.032 |
| **N stage**  N0  N1  N2  N3 | 1  1  1  1 | 0.17(0.12-0.23)  0.29(0.19-0.44)  0.47(0.21-1.05)  0.25(0.09-0.70) | <0.001  <0.001  0.065  0.008 | 0.22(0.18-0.27)  0.37(0.30-0.46)  0.41(0.20-0.81)  0.14(0.05-0.40) | <0.001  <0.001  0.011  <0.001 | 0.13(0.10-0.18)  0.21(0.16-0.27)  0.20(0.10-0.43)  0.15(0.05-0.47) | <0.001  <0.001  <0.001  0.001 | 0.50(0.40-0.64)  0.61(0.45-0.83)  0.48(0.20-1.12)  1.56(0.37-6.62) | <0.001  0.001  0.089  0.54 | 0.34(0.22-0.52)  0.56(0.39-0.81)  0.42(0.16-1.15)  0.18(0.04-0.94) | <0.001  0.002  0.092  0.042 | <0.001 |
| **Tumor location**  Upper third  Middle third  Lower third  Unknown | 1  1  1  1 | 0.39(0.19-0.78)  0.24(0.14-0.41)  0.25(0.19-0.33)  0.22(0.10-0.50) | 0.008  <0.001  <0.001  <0.001 | 0.26(0.16-0.40)  0.21(0.15-0.30)  0.32(0.270.39)  0.33(0.22-0.50) | <0.001  <0.001  <0.001  <0.001 | 0.04(0.01-0.30)  0.13(0.08-0.21)  0.19(0.15-0.24)  0.11(0.05-0.25) | 0.002  <0.001  <0.001  <0.001 | 0.50(0.28-0.88)  0.37(0.25-0.55)  0.65(0.51-0.83)  0.56(0.30-1.02) | 0.016  <0.001  <0.001  0.06 | 0.28(0.10-0.80)  0.37(0.20-0.67)  0.51(0.36-0.71)  0.34(0.18-0.66) | 0.018  0.01  <0.001  0.001 | 0.13 |
| **Marital status**  Married  Unmarried  Unknown | 1  1  1 | 0.24(0.18-0.33)  0.28(0.20-0.38)  0.14(0.05-0.40) | <0.001  <0.001  <0.001 | 0.27(0.22-0.33)  0.31(0.25-0.38)  0.38(0.21-0.70) | <0.001  <0.001  0.002 | 0.16(0.13-0.21)  0.16(0.12-0.22)  0.30(0.14-0.66) | <0.001  <0.001  0.002 | 0.48(0.67-0.63)  0.61(0.47-0.78)  0.58(0.26-1.32) | <0.001  <0.001  0.20 | 0.41(0.29-0.57)  0.54(0.34-0.88)  0.35(0.14-0.83) | <0.001  0.012  0.018 | 0.66 |
